# A TLR7/8 Agonist-Including DOEPC-Based Cationic Liposome Formulation Mediates Its Adjuvanticity Through the Sustained Recruitment of Highly Activated Monocytes in a Type I IFN-Independent but NF-κB-Dependent Manner

**DOI:** 10.3389/fimmu.2020.580974

**Published:** 2020-11-11

**Authors:** Floriane Auderset, Elodie Belnoue, Beatris Mastelic-Gavillet, Paul-Henri Lambert, Claire-Anne Siegrist

**Affiliations:** World Health Organization Collaborating Center for Vaccine Immunology, Departments of Pathology-Immunology and Pediatrics, University of Geneva, Geneva, Switzerland

**Keywords:** liposome, TLR7/8 agonist, germinal centers, follicular T helper cells, adjuvants for vaccine

## Abstract

Novel adjuvants, such as Toll-like receptors (TLRs) agonists, are needed for the development of new formulations able to circumvent limitations of current vaccines. Among TLRs, TLR7/8 agonists represent promising candidates, as they are well described to enhance antigen-specific antibody responses and skew immunity toward T helper (T_H_) 1 responses. We find here that the incorporation of the synthetic TLR7/8 ligand 3M-052 in a cationic DOEPC-based liposome formulation shifts immunity toward T_H_1 responses and elicits strong and long-lasting germinal center and follicular T helper cell responses in adult mice. This reflects the prolonged recruitment of innate cells toward the site of immunization and homing of activated antigen-loaded monocytes and monocyte-derived dendritic cells toward draining lymph nodes. We further show that this adjuvanticity is independent of type I IFN but NF-κB-dependent. Overall, our data identify TLR7/8 agonists incorporated in liposomes as promising and effective adjuvants to enhance T_H_1 and germinal center responses.

## Introduction

Vaccines play a key role against infectious diseases such as smallpox, diphtheria, tetanus, measles and many others. The unavailability of vaccines for other diseases, or vaccines whose protective efficacy is limited in magnitude or duration, reflect various challenges such as poor immunogenic properties of the antigens, recipient populations with less-responsive immune system, lack of defined correlates of protection or safety (1). Some were met by the use of more potent adjuvants than the aluminium salts (Alum) used in most current vaccines (2). A striking recent example is the use of AS01_B_ in the recombinant adjuvanted zoster vaccine (Shingrix^®^, GlaxoSmithKline), whose adjuvant effect depends on the synergy of the QS21 saponin and the Toll-like receptor (TLR)-4 agonist Monophosphoryl lipid A (MPL) (3, 4).

TLRs can sense a broad range of microbial components, are widely expressed by innate cells and play a crucial role in shaping both innate and adaptive immune responses of the host upon pathogen infection. Among the different TLRs, the endosomal TLR7 and TLR8 have been of particular interest since both are expressed by the major human dendritic cell (DC) subsets (plasmacytoid and myeloid DCs, respectively) (5) and TLR7 by human B cells (6, 7). Both are activated by ssRNA as well as small synthetic molecules such as imidazoquinolines, which can be dual TLR7/8 agonists (e.g. resiquimod (R848)) or TLR7-selective (e.g. imiquimod (R837)) (8).

*In vitro*, TLR7/8 agonists have been shown to enhance maturation, costimulatory marker expression and cytokine secretion of human DCs (9), as well as cellular and humoral adaptive responses (10), prompting their evaluation as vaccine adjuvants mostly in animal models, but also in humans (11). However, the strong effect of TLR7 *in vitro* stimulation has been difficult to translate *in vivo* in murine models. While some studies reported the development of a T helper (T_H_) 1 adaptive immunity, characterized by the increased induction of IFN-γ-secreting CD4^+^ T cells, cytotoxic CD8^+^ T cell responses and IgG2-producing B cells (12–15), others failed to demonstrate an effect (16–18) or showed only a modest impact (17, 19–22). This reflected mostly the solubility and small size of the molecules, resulting into their rapid diffusion in circulation and into systemic pro-inflammatory cytokine release (17, 21, 23), thus limiting the use of TLR7/8 ligands to topical administration rather than parenteral application (24).

To bypass systemic toxicity and to increase adjuvanticity, efforts have been made to develop formulations with TLR7/8 agonists with physicochemical properties that promote their retention at the injection site (25–28). The lipid tail of the imidazoquinoline 3M-052 TLR7/8 agonist, for example, allows its gradual delivery when formulated in oil-in-water emulsions or incorporated into the lipid bilayer of liposomes, preventing the systemic release of TNF-α and detection of pro-inflammatory cytokines in the spleen (26, 27). When formulated in liposomes, 3M-052 enhanced T_H_1 adaptive immunity to hemagglutinin antigen (HA) (27) and HBsAg (29) and protected mice and ferrets from lethal H5N1 homologous challenge (30) through the induction of IgG2a antibodies (Ab) and IFN-γ secretion, whereas soluble R848 was ineffective (17, 27). It also promoted functional CD8^+^ T cell responses to OVA antigen (31). When adsorbed to alum, 3M-052 enhanced Ab responses and T_H_1 cellular responses to tuberculosis and HIV antigens (32), and markedly enhanced Ab responses, B cell activation and generation of Ag-specific CD4^+^ T_H_1 cells to the alum-based pneumococcal conjugate vaccine Prevnar13^®^ in a neonatal rhesus macaque model (33).

In this study, we used the glycoprotein B (gB) of cytomegalovirus (34) as a model antigen to investigate the mechanisms by which a DOEPC-based cationic liposome formulation including the 3M-052 TLR 7/8 agonist (hereafter named SPA10) exerts its adjuvanticity in comparison to the plain DOEPC-based cationic liposome formulation (hereafter named SPA06), used as control. We show that strong and sustained germinal center (GC) B cell and T follicular helper (T_FH_) cell responses are induced by SPA10, associated with a prolonged recruitment/homing of highly activated antigen-loaded monocytes and monocyte-derived dendritic cells (Mo-DCs) toward the injection site and draining lymph nodes (dLNs), and that SPA10-mediated B cell adjuvanticity is type I interferon-independent but NF-κB-dependent.

## Material and Methods

### Mice

C57BL/6 mice were purchased from Charles River (L’Arbresle, France). IFNAR^-/-^ and NF-κB^-/-^ mice on a C57BL/6 background were purchased from The Jackson Laboratory (Ellsworth, United States). All mice were bred and maintained under specific pathogen free conditions. Mice were used at 6-8 weeks of age. All animal experiments were carried out in accordance with Swiss and European guidelines and approved by the Geneva Veterinary Office under the authorization number 1005/3610/1, G05/3885/1 and GE/2/15.

### Antigens, Adjuvants, and Immunizations

Mice were immunized intramuscularly (i.m.) or intraperitoneally (i.p.), with 2 μg of gB-CMV [SANOFI Pasteur, prepared as described in (34)], adjuvanted with cationic liposomal formulations (SANOFI Pasteur) including 100 μg 1,2-dioleoyl-*sn*-glycero-3-ethylphosphocholine (chloride salt) (DOEPC; Avanti Polar Lipids, Alabaster, AL, USA) without (SPA06) or with (SPA10) 17.5 μg 3M-052, in a final volume of 50 μl. These doses were each experimentally defined as optimal in preliminary dose optimization studies. Due to its lipophilic nature, 3M-052 was tightly bound to DOEPC (at a molar ratio of DOEPC/3M052 of 4:1) in the SPA10 formulation. SPA06 and SPA10 (SPA being the acronym for Sanofi Pasteur Adjuvant) were produced by using a solvent removal technique to produce liposomes in a 15% sucrose solution. The resulting formulations were passed through a 0.22 µm filter membrane, filled in type I glass vials, lyophilized, and capped and sealed under nitrogen. At the time of immunization, the lyophilizates containing 2 mg of DOEPC (SPA06) or 2 mg of DOEPC plus 0.35 mg of 3M-052 (SPA10) were first reconstituted with 0.5 ml of water for injection prior the addition of 0.5 ml of gB-CMV (at 80 µg/ml in CMV buffer) under aseptic conditions. After 30 min of incubation, the resulting product was used without further characterization for the immunization of mice. Although the interaction between gB-CMV and SPA06 or SPA10 was not specifically characterized, gB-CMV, which displays a net negative charge at pH 7.0 (pI=4), was expected to interact with the cationic liposomes through electrostatic interactions. Before the addition of gB-CMV, and after rehydration, SPA06 and SPA10 displayed an average particle size of 96 nm and 172 nm with a polydispersity index of 0.43 and 0.5 (Zetasizer N4; Malvern Instrument; Malvern, UK), respectively. For antigen and adjuvant tracking experiments, gB-CMV was conjugated to Alexa fluor 647 while SPA06 and SPA10 were fluorescently labeled with DiO dyes. Fluorescent labeling of SPA06 and SPA10 was achieved by mixing DiO into the starting lipid solution at a ratio of 0.5 or 1.0% with respect to total lipid (w/w ratio). CMV-gB was fluorescently labeled by using the Alexa Fluor^®^633 Protein Labeling Kit from Molecular Probes (ThermoFisher) according to manufacturer’s instructions.

### Quantification of Antibodies

gB-CMV-specific antibody titers were determined by Ag-capture ELISAs. Briefly, 96-well plates were coated with gB-CMV (1 μg/ml). Wells were incubated with 1:2 serial dilutions of individual mouse sera prior to incubation with secondary horseradish peroxidase (HRP)-conjugated anti-mouse IgG (Invitrogen, Life technologies), IgG1 (BD Pharmingen) and IgG2c (Southern Biotech). The optical density of each well was measured at 405 nm and the data analyzed with SoftMax Pro software. Results are expressed as Log10 of gB-specific titers determined by reference to serial dilutions of a titrated pool of hyperimmune sera.

### Antigen-Specific T Cell Response

Mice were boosted at d21 post prime, and spleens were harvested 14 days post boost. Five million cells were cultured at 37°C in 48-well plates, in 500 μl of 10% FCS-containing DMEM (Gibco) with/without gB-CMV (5 μg/ml). Cell-free supernatants were collected after 72h for IFN-γ and IL-5 quantification by capture ELISA. Briefly, plates were coated with purified mouse anti-IFN-γ (BD Biosciences) or anti-IL-5 (BD Biosciences) capture Abs. Serial dilutions of supernatants were added prior to incubation with biotinylated mouse anti-IFN-γ (eBioscience) and IL-5 (BD Biosciences), respectively, followed by HRP-conjugated streptavidin (Roche). Optical densities were measured at 405nm and data analyzed with SoftMax Pro. Results were expressed by reference to a standard curve included in each plate.

### Flow Cytometric Analysis

For GC and T_FH_ cell analysis, dLNs were harvested at the indicated time-points post immunization. Single cell suspensions were stained with the following Abs: CD4-Pacific Blue (clone GK1.5) (BioLegend); GL7-FITC (cloneGL7); CD8-APC-Cy7 (clone53-6.7); CD11b-biotin (clone MI/70); B220-PE Texas Red, -PE-CF594 (clone RA3-6B2) (all from BD Biosciences); PD-1-PE (clone J43); Gr-1-biotin (clone RB6-8C5); Ter119-biotin (clone Ter119); B220-PerCP-Cy5.5 (clone RA3-6B2); TCRβ-PE (clone H57-597); Fas-biotin (clone 15A7) (all from eBioscience). CXCR5 was stained using a purified anti-mouse CXCR5 (BD Bioscience), followed by a goat anti-rat IgG-FITC (Southern Biotech). Biotinylated antibodies were revealed with Alexa fluor 700- (Molecular Probe) or APC-conjugated streptavidin (BD Biosciences). For adjuvant/antigen tracking experiments, dLNs were harvested, cut in small pieces and digested with a Pasteur pipette for 10-15’ with 1 μg/ml liberase CI (Roche) and 1 mg/ml DNase (Sigma) in serum-free DMEM (Gibco). Muscles were cut in small pieces and digested at 37°C for 30 min in HBSS medium (Gibco) supplemented with 0.5 mg/ml Collagenase Type II (Worthington) and 0.5 mg/ml DNase (Sigma). Single cell suspensions were filtered and stained with the following Abs: Ly6G-PE (clone 1A8); B220-Pacific Blue (clone RA3-6B2); Ly6C-Alexa fluor700 (clone AL-21); CD86-PE (clone GL-1) (all from BD Biosciences); CD11c-PE-Cy7 (clone N418); CD11b-APC-Cy7 (clone M1/70); (all from BioLegend); CD80-APC (clone 16-10A1) (all from eBioscience). Data were acquired on a Gallios flow cytometer (Beckman Coulter) and analyzed using FlowJo Software (Tree Star).

### IFN-α Detection in Intraperitoneal Lavages

Peritoneal lavages were performed 12 h post i.p. immunization with 1 ml of 2% FCS-containing PBS. Lavage was recovered with a glass Pasteur pipette and cells were removed by centrifugation. IFN-α was measured in the supernatant by a commercial ELISA kit (pbl Assay Science) following the supplier’s instructions.

### Real-Time PCR

Harvested muscles and dLNs of immunized mice were snap-frozen in liquid nitrogen and were polytroned in RLT lysis buffer (Qiagen). Total mRNA was extracted using RNeasy mini kit (Qiagen), according to manufacturer’s protocol and 0.5mg of mRNA was used to synthetize cDNA using a mix of random hexamers, oligo d(T) primers and PrimerScript II reverse transcriptase enzyme (PrimeScript II 1st strand cDNA Synthesis Kit; Takara). Semi quantitative real-time PCR were performed using SYBR Green PCR Master Mix (Applied Biosystems) and 300nM forward and reverse primers on a SDS 7900 HT instrument (Applied Biosystems). Each sample was tested in triplicate and normalized to the endogenous expression of *actin G*, *EEf1* and *GAPDH* control genes. Data normalization was calculated using the GeNorm method. Primers used were as follow: *stat1*-forward 5’-GCCTGGATCAGCTGCAAAG-3’ and -reverse 5’-GCTGCAGGGTCTCTGCAAC-3’; *stat2*-forward 5’-GAGTTACTTCAGCGTCTGCTCCA-3’ and -reverse 5’-GGGCTGGGTTTCTACTACAAAGG-3’; *irf9*-forward 5’-TGGAGCATCAACTTCCTCTGAA-3’ and -reverse 5’-TGAAGGTGAGCAGCAGCG-3’; *actin G, EEf1, GAPDH* (35); and *IFN-α*, *IRF-7*, *MyD88*, *TLR7* (36) as previously described.

### Statistical Analysis

Data were analyzed using the Mann-Whitney U test for unpaired data. When more than two groups were tested, the statistical analysis was performed using a 1-way ANOVA followed by a Tukey multiple comparison test. Differences with p > 0.05 were considered insignificant.

## Results

### SPA10 Adjuvantation Switches Primary Antibody Responses From IgG1 to IgG2c

**Figure 1:**
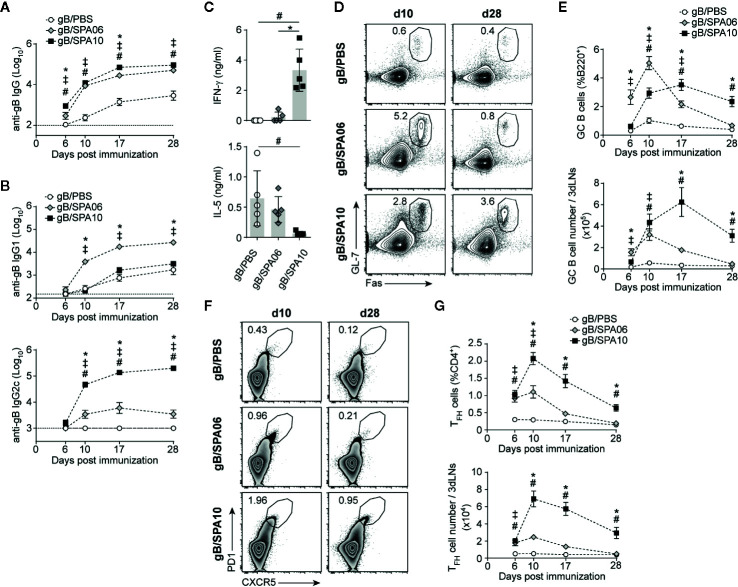
Liposomal-based vaccines elicit strong T_FH_ and germinal center (GC) B cell responses, which are further increased by TLR7/8 ligands. **(A, B)** Naïve C57BL/6 mice were immunized once intramuscularly (i.m.) in both hind legs and euthanized at days 6, 10, 17 and 28 p.i. Serum levels of gB-specific **(A)** IgG and **(B)** IgG1 and IgG2c were assessed by ELISA. Results are expressed as mean ± SEM obtained from three independent experiments with n = 3–4 mice per group each. **(C)** Mice were immunized i.m. with gB/PBS, gB/SPA06 and gB/SPA10 and boosted at day 21. Spleens were harvested at day 35, and IL-5 and IFN-γ secretion was quantified in supernatants of splenocytes restimulated or not with gB for 72h. The mean cytokine level by gB-stimulated cells after subtraction of medium condition ± SEM are given for n = 5 mice per group. One out of 2 independent experiment is shown. **(D–G)** Inguinal and para-aortic draining lymph nodes were harvested at indicated time-points after a single immunization with gB/PBS, gB/SPA06 or gB/SPA10, and analyzed by flow cytometry for the presence of GC and T_FH_ cells. Representative contour and zebra plots, respectively, show **(D)** the GL7^+^Fas^+^ GC B cell population gated on B220^+^ and **(F)** the CXCR5^+^PD1^+^ T_FH_ cell population gated on CD4^+^ T cells at days 10 and 28 p.i. Numbers in plots represent the frequency of the gated population. The mean frequency and number of **(E)** GC B cells and **(G)** T_FH_ cells ± SEM from three independent experiments with n = 3–4 mice per group are given. ^‡^ *p* < 0.05 gB/PBS vs gB/SPA06; ^#^ *p* < 0.05 gB/PBS vs gB/SPA10; *p < 0.05 gB/SPA06 vs gB/SPA10.

Adult C57BL/6 mice were immunized once i.m. with 2 μg of the cytomegalovirus glycoprotein B (gB) admixed to cationic liposomes without (SPA06) or with (SPA10) 17.5 μg of 3M-052 TLR 7/8 agonist, or in PBS (control). Both adjuvants strongly enhanced gB-specific IgG responses compared to PBS, as early as 6 days post immunization (p.i.) (**Figure 1A**). SPA10 significantly but only slightly further increased gB-specific IgG levels compared to SPA06 (**Figure 1A**). As expected from previous studies (21, 27, 37), the presence of the TLR 7/8 agonist shifted Ab responses from IgG1 toward IgG2c (**Figure 1B**). Accordingly, gB-stimulated splenocytes isolated from gB/SPA10-immunized mice secreted significantly more IFN-γ compared to splenocytes from gB/PBS- or gB/SPA06-immunized mice, while their secretion of IL-5 was almost abrogated (**Figure 1C**). This demonstrates the potent adjuvanticity of both the SPA06 and SPA10 cationic liposomes on B cell responses - and the ability of SPA10 to shift such responses from T_H_2 toward T_H_1 preferential responses.

### SPA10 Elicits Stronger and Longer-Lasting GC B and T_FH_ Cell Responses

Cationic liposomes mainly enhance B cell responses by increasing the magnitude of germinal centers (GC), both in term of duration and number of GC B cells as compared to responses elicited by non-adjuvanted antigen (38), resulting in the production of high affinity antibodies by long-lived plasma cells and increased memory B cells (39, 40). We thus investigated by flow cytometry the influence of SPA06 and SPA10 on GC formation in the inguinal and iliac draining lymph nodes (dLNs) following immunization. gB/PBS elicited a modest and transient GC response, peaking at day 10 and terminated by day 17. SPA06 significantly increased GC responses, with the same kinetics. GC responses to SPA10 were more delayed but remarkably longer-lasting, as reflected by significantly lower frequencies and numbers of GL-7^+^Fas^+^ GC B cells in gB/SPA10-immunized mice on day 6 and 10 but higher frequencies on day 28 (last time point assessed, **Figures 1D, E**).

As T_FH_ cells are essential to GC development and maintenance (41), we assessed the influence of SPA06 and SPA10 on their induction. SPA10 significantly increased both the frequency and number of CD4^+^PD1^high^CXCR5^+^ T_FH_ cells compared to SPA06 from day 6 onwards, with a peak response on day 10. Again, SPA10 maintained T_FH_ cell responses up to 28 days p.i. (last time point assessed), whereas T_FH_ cells elicited by SPA06 had returned to baseline by day 28 (**Figures 1F, G**). Altogether, these data show that SPA06 and SPA10 both markedly enhance GC B and T_FH_ cell responses, the TLR7/8-containing SPA10 formulation significantly prolonging their duration and further enhancing T_FH_ cell numbers.

### SPA10 Sustains the Recruitment of Innate Cells Toward the Site of Immunization

The recruitment of innate immune cells (such as neutrophils, dendritic cells (DCs), monocytes and others) to the site of immunization is critical for the priming of adaptive immune responses. We postulated that the extended action of SPA10 versus SPA06 on GC B and T_FH_ cells may result from differences in innate cell recruitment at the injection site.

**Figure 2:**
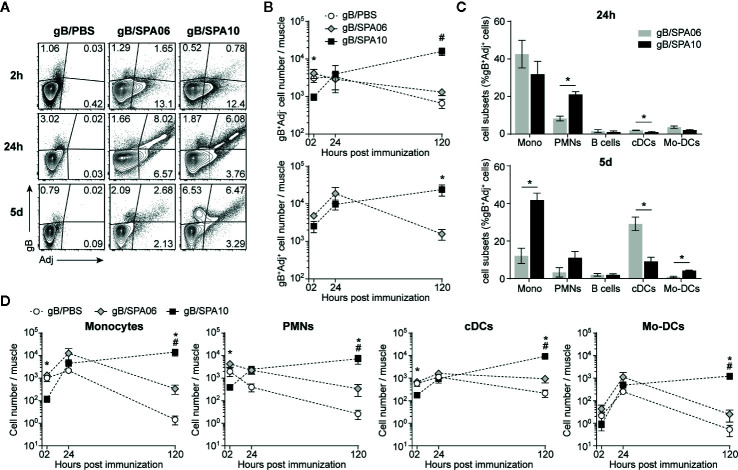
SPA10 induces sustained innate cell recruitment at the site of immunization. Naïve C57BL/6 mice were immunized i.m. in both hind legs with fluorescently labeled gB-Alexa633 and SPA06-DiO or SPA10-DiO or PBS as control. Muscles were collected after 2 h, 24 h, and 5 days and individually analyzed by flow cytometry after enzymatic digestion. **(A)** Representative dot plots display the frequency of gB-, SPA06-, or SPA10-loaded cells within lymphocyte population after doublets exclusion. **(B)** Graphs show the mean number per muscle of gB^+^Adj^-^ (top graph) or gB^+^Adj^+^ (bottom graph) cells within lymphocytes ± SEM. **(C)** Histograms show the mean frequency of several innate cell types identified by specific markers among gB^+^SPA06^+^ and gB^+^SPA10^+^ cell populations in muscles 24 h and 5 days post immunization (monocytes:CD11c^-^ CD11b^+^ Ly6G^-^ Ly6C^high^; PMNs: CD11c^-^ CD11b^+^ Ly6G^+^ Ly6C^int^; B cells: CD11c^-^ CD11b^-^ B220^+^; cDCs: CD11c^+^ CD11b^+/-^ Ly6C^-^; Mo-DCs: CD11c^+^ CD11b^+^ Ly6C^+^) ± SEM. **(D)** The kinetics of different total cell population recruitment are shown. Mean cell number ± SEM for each population is given. All results are obtained from three independent experiments with n = 2–3 mice per group each. ^#^ *p* < 0.05 for gB/PBS vs gB/SPA10; *p < 0.05 gB/SPA06 vs gB/SPA10.

To compare the innate immune cell patterns, we immunized mice i.m. with fluorescently-labelled gB (gB-Alexa647) in SPA06 or SPA10 liposomes marked with the lipophilic fluorescent dye DiO (or in PBS). In the injected muscles, cells loaded with gB, free or within liposomes, were readily detected in all groups at 2, 24, and 120 h p.i. (**Figure 2A**). SPA06 did not affect gB uptake compared to PBS, with maximum levels detected at 2h post-injection and progressive decline thereafter (**Figures 2A, B**). SPA10 delayed gB uptake, as reflected by the significantly lower number of gB^+^ Adjuvant (Adj)^-^ cells and slightly lower number of gB^+^Adj^+^ cells detected after 2 h. After 24 h, similar numbers of gB^+^Adj^-^ and gB^+^Adj^+^ cells were found in muscles of mice immunized with gB/SPA10 and gB/SPA06 (**Figures 2A, B**). The number of gB^+^Adj^-^ and gB^+^Adj^+^ cells further increased only in mice immunized with gB/SPA10, up to 5 days p.i. (last time-point assessed).

As gB^+^Adj^-^ cells represent only a marginal population as compared to double positive cells, we next characterized gB^+^Adj^+^ cells in the injected muscle 24h post immunization (**Figure 2C**). gB^+^Adj^+^ cells mainly comprised monocytes (Mono; CD11c^-^ CD11b^+^ Ly6G^-^ Ly6C^high^) and neutrophils (PMNs; CD11c^-^ CD11b^+^ Ly6G^+^ Ly6C^int^), which were significantly more numerous in gB/SPA10- versus gB/SPA06-immunized mice. Conventional DCs (cDCs; CD11c^+^ CD11b^+/-^ Ly6C^-^) and monocyte-derived DCs (Mo-DCs; CD11c^+^ CD11b^-^ Ly6C^+^) were only marginally represented within gB^+^Adj^+^ cells at this time-point (**Figure 2C**). Five days after immunization, gB^+^SPA10^+^ cells still included a majority of monocytes, followed by PMNs, cDCs and Mo-DCs, reflecting sustained local inflammation. In contrast, we observed a shift between monocytes (~10% of gB^+^Adj^+^ cells) and cDCs (~30% of gB^+^Adj^+^ cells) in mice immunized with gB/SPA06 (**Figure 2C**).

These data suggest that SPA10 – but not SPA06 - prolongs the recruitment of innate cells toward the site of immunization. This was confirmed by assessing the number of total monocytes, PMNs, cDCs and Mo-DCs in the injected muscles. Recruitment of inflammatory monocytes, PMNs, cDCs and Mo-DCs was slightly, but not-significantly, increased by SPA06 and followed similar kinetics to PBS. In contrast, SPA10 altered the kinetics of innate cell recruitment. Initially significantly lower at 2 h p.i., the number of recruited innate cells increased over time to reach comparable levels to SPA06 at 24h, and kept increasing up to 5 days p.i. (last time-point assessed, **Figure 2D**). Consistently, the number of gB^+^Adj^+^ cells (**Figure 2B**) followed similar kinetics, confirming that the TLR7/8-containing SPA10 cationic liposome formulation triggers a sustained recruitment of a variety of innate cells toward the site of injection.

### Influence of the Formulation on the Influx of Activated Antigen-Presenting Cells in the dLNs

**Figure 3:**
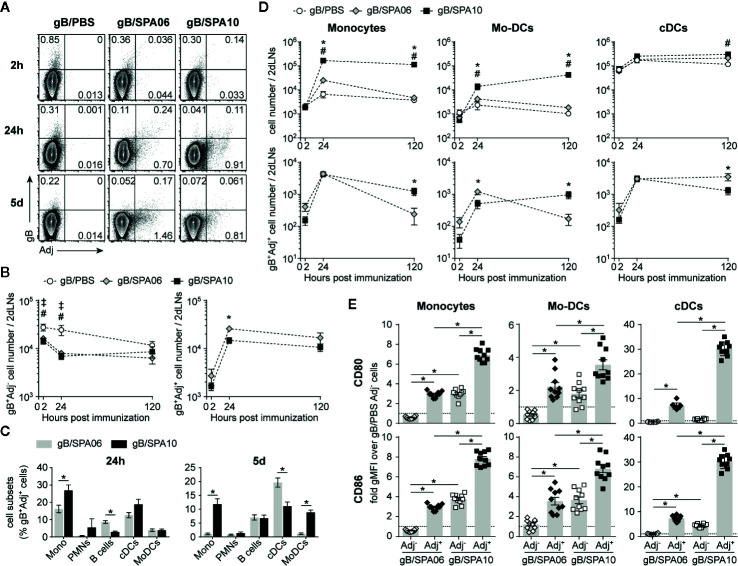
SPA10 induces sustained influx of Ag-loaded cells into the draining lymph nodes (dLNs). **(A–D)** naïve C57BL/6 mice were immunized i.m. in both hind legs with fluorescently labeled gB-Alexa633 and SPA06-DiO or SPA10-DiO or PBS as control. Draining LNs from each mouse side (inguinal and para-aortic) were collected after 2 h, 24 h, and 5 days and separately analyzed by flow cytometry after enzymatic digestion. **(A)** Representative dot plots display the frequency of gB-, SPA06-, or SPA10-loaded cells within lymphocyte population after doublets exclusion. **(B)** The mean number per two dLNs (para-aortic + inguinal) of gB^+^Adj^-^ (top graph) or gB^+^Adj^+^ (bottom graph) cells within lymphocytes ± SEM is shown. **(C)** Histograms show the mean frequency of various innate cell types among gB^+^SPA06^+^ and gB^+^SPA10^+^ cell populations (defined as in Figure 2) 24 h and 5 days post immunization ± SEM. **(D)** Graphs on the top represent the kinetics of innate cell recruitment with the mean cell number ± SEM for each population. Graphs on the bottom show the mean cell number of gB^+^Adj^+^ cells for each sub-population ± SEM. All results are obtained from two independent experiments with n = 4 dLNs per group each (2 mice). **(E)** Draining LNs from each mouse side (inguinal + para-aortic) were separately treated by enzymatic digestion 24h post i.m. immunization with gB/PBS, gB/SPA06-DiO or gB/SPA10-DiO in both hind legs and CD80 and CD86 expression was assessed on Adj^-^ and Adj^+^ monocytes, Mo-DCs and cDCs (identified as in **Figure 2**) by flow cytometry. The fold-change of geometrical mean fluorescence intensity over PBS (dotted line) is shown ± SEM for n = 10 dLNs per group (5 mice). ^‡^ *p* < 0.05 for gB/PBS vs gB/SPA06; ^#^ *p* < 0.05 for gB/PBS vs gB/SPA10; *p < 0.05 gB/SPA06 vs gB/SPA10.

We then asked whether the differences in innate cell recruitment pattern observed at the site of injection recapitulate in draining lymph nodes, thereby extending the action of SPA10 on GC B and T_FH_ cell responses. A population of gB-loaded cells was already present in the dLNs 2h after injection in the three groups of mice, a time at which the liposomes were barely detectable (**Figure 3A**), suggesting early self-drainage of the gB antigen to the dLNs. Adjuvantation with SPA06 or SPA10 both reduced the number of gB^+^Adj^-^ cells reaching the dLNs, indicating antigen retention at the site of injection (**Figure 3B**). gB^+^Adj^+^ cells appeared in the dLNs between 2 and 24 h and numbers plateaued up to 5 days, at similar level following adjuvantation with SPA06 or SPA10 (**Figures 3A, B**).

At 24 h p.i., gB^+^SPA06^+^ cells included cDCs, few monocytes and many B cells (**Figure 3C**). In contrast, monocytes and cDCs predominated within gB^+^SPA10^+^ cells. On day 5, gB^+^SPA06^+^ cells included cDCs and B cells while gB^+^SPA10^+^ cells maintained a broader distribution of cells, including monocytes, B cells, cDCs and Mo-DCs (**Figure 3C**), reflecting the cell recruitment observed at the injection site (**Figure 2C**). We next analyzed the number of total and gB^+^Adj^+^ cells in dLNs. PMNs being only marginally represented within gB^+^Adj^+^ cells (**Figure 3C**), we focused our analyses on monocytes, Mo-DCs and cDCs. Again, SPA06 did not increase the recruitment of innate cells to the dLNs compared to PBS (**Figure 3D**, top panel). In contrast, a significantly higher number of monocytes and Mo-DCs, but not of cDCs, migrated toward the dLNs following immunization with SPA10. As observed in the injection-site muscles (**Figure 2D**), the number of monocytes and Mo-DCs remained elevated up to 5 days (last time-point assessed) post immunization with gB/SPA10. The number of gB- and Adj-loaded monocytes were comparable at 24h p.i., with higher numbers of Mo-DCs in the dLNs of gB-SPA06- than SPA10-injected mice. However, 5 days after immunization, gB^+^Adj^+^ monocytes and Mo-DCs (**Figure 3D**, lower panel) were higher only in dLNs from SPA10-immunized mice.

We also investigated whether monocytes, Mo-DCs and cDCs migrating toward dLNs were differentially activated following SPA06 and SPA10 adjuvantation. As the vast majority of gB-loaded cells were Adj^+^ cells (**Figure 3A**), we compared the levels of CD80 and CD86 expression of Adj^+^ and Adj^-^ cells versus PBS. SPA06^+^ and SPA10^+^ monocytes, Mo-DCs and cDCs expressed higher levels of both CD80 and CD86 than SPA06^-^ cells which did not show detectable levels of activation (**Figure 3E**). gB^+^SPA06^-^ cells expressed the same levels of CD80/CD86 as control gB^+^/PBS cells. However, gB^+^SPA10^-^ monocytes and Mo-DCs expressed much higher levels of CD80 and CD86, similarly to SPA06^+^ cells (**Figure 3E**). Thus, both SPA06 and SPA10 adjuvants induce the migration of gB-loaded innate cells toward the dLNs, suggesting that the presence of TLR7/8 agonist in the formulation is associated with sustained recruitment of activated monocytes and Mo-DCs that comprise Ag-loaded and activated Ag^-^ bystander cells in the vicinity.

### SPA10 Induces Functional Type I IFN Responses Via TLR7 Signaling

**Figure 4:**
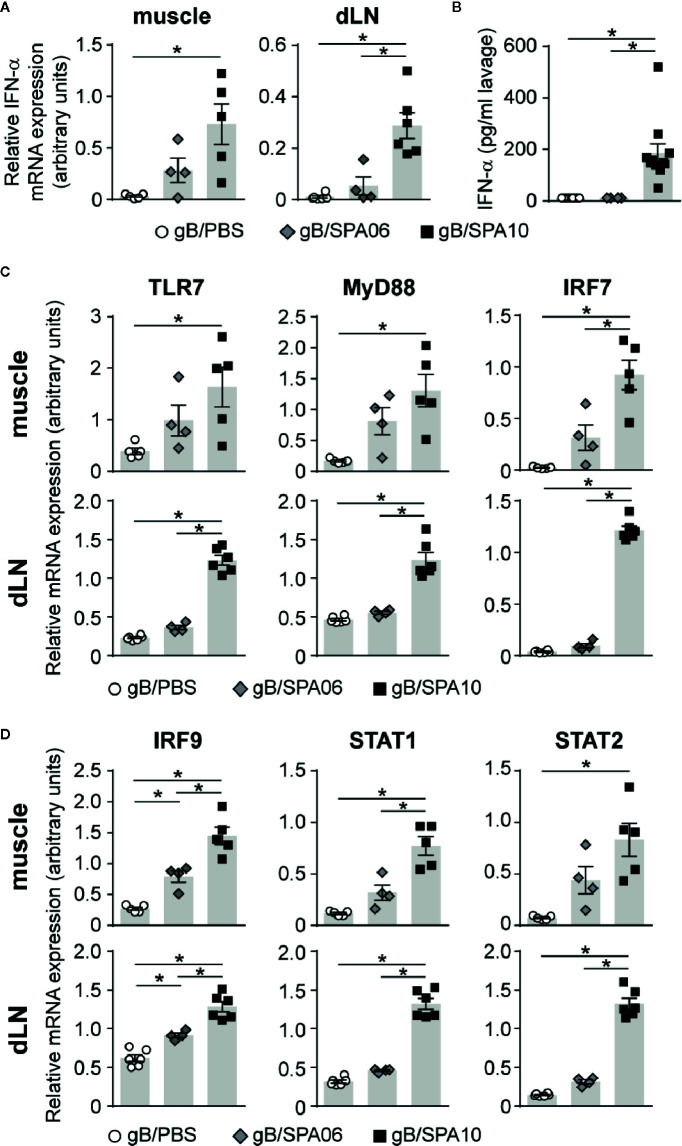
SPA10 induces a potent type I IFN response in adult mice. Naïve C57BL/6 mice were immunized **(A, C, D)** i.m. or **(B)** intraperitoneally with gB/SPA06, gB/SPA10 or gB/PBS as control. The mRNA expression levels of **(A)** IFN-α, **(C)** TLR7, MyD88, IRF7 and **(D)** IRF9, STAT1, and STAT2 were assessed by semi-quantitative real-time PCR in whole dLNs and muscles after 12 h and normalized to three constitutively expressed control genes. Results are represented as arbitrary units ± SEM for n ≥ 3 mice per group. One of two independent experiments is shown. **(B)** IFN-α was measured by ELISA in cell-free supernatants of peritoneal lavages of immunized mice performed 12 h post i.p. immunization. The mean IFN-α concentration ± SEM from two independent experiments with n ≥ 3 mice per group is given. *p < 0.05.

The ligation of its agonist to TLR7/8 induces phosphorylation of IRF7 *via* the adaptor protein MyD88, resulting in the induction of type I IFN responses and expression of IFN-α (42). To define whether the adjuvanticity of SPA10 was mediated *via* the TLR7 pathway, we assessed the mRNA expression of several TLR7/8 signaling-related genes in injected muscles and dLNs by RT-PCR. A strong increase in IFN-α mRNA expression was observed in muscles and dLNs 12 p.i. (**Figure 4A**) – but not earlier (not shown), together with a higher expression of TLR7, MyD88 and IRF7 (**Figures 4C**) compared to gB/PBS-immunized mice. SPA06 induced a limited increase in IFN-α, TLR7, MyD88 and IRF7 expression in the injected muscles, and not in the dLNs (**Figures 4A, C**). To validate our observations of the induction of IFN-α at the injection site and circumvent the tricky detection of INF-α in the muscle (not shown), we quantified IFN-α by ELISA in the peritoneal fluid 12 h following intra-peritoneal immunization. Only gB/SPA10 significantly increased IFN-α levels (**Figure 4B**).

Upon binding of IFN-α to the IFNAR receptor, phosphorylated STAT1 and STAT2 form a complex with IRF9, which further translocates into the nucleus to induce the expression of IFN-inducible genes (42). To determine whether SPA10 exerts its adjuvanticity *via* type I IFN signaling, we assessed the mRNA expression of IFNAR pathway-related genes. Indeed, gB/SPA10 significantly upregulated IRF9, STAT1 and STAT2 mRNA expression, while gB/SPA06 induced intermediate levels of these molecules, with significant differences only detected for IRF9 as compared to gB alone (**Figure 4D**). Altogether, these data indicate that the incorporation of 3M-052 in cationic liposomes enhances type I IFN responses *via* TLR7 signaling, both at the site of injection and in the dLNs, while the SPA06 liposomes only exert a limited type I IFN inflammatory reaction at the injection site.

### gB/SPA10 Adjuvanticity Is Independent of Type I IFN Response

**Figure 5:**
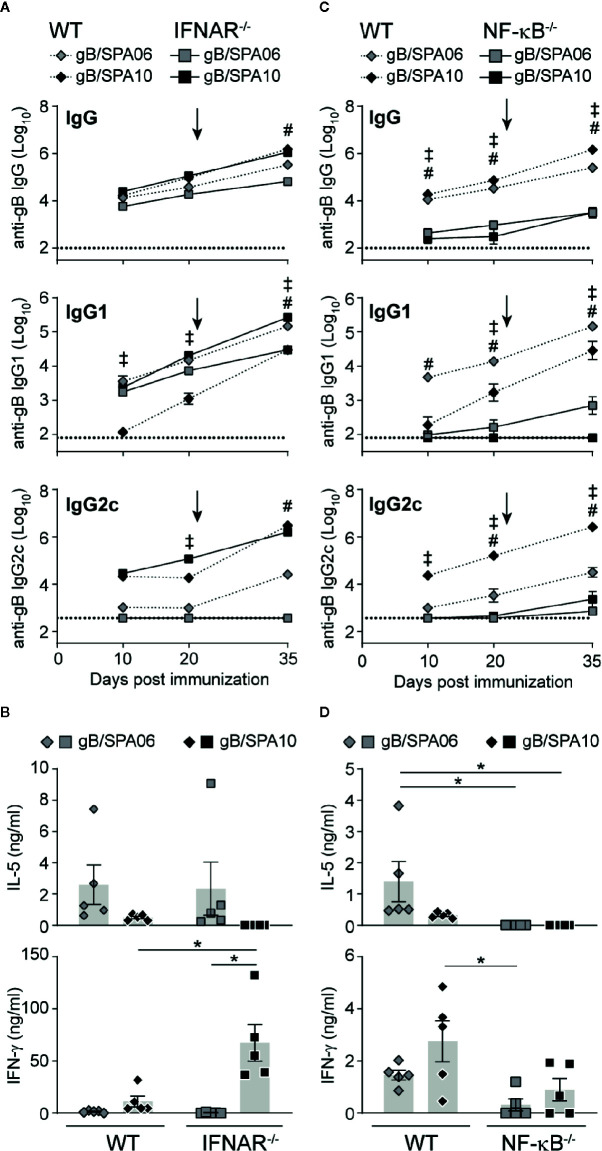
SPA10 immune responses are type I IFN-independent and NF-κB-dependent. Naïve WT C57BL/6 (dashed lines) and **(A, B)** IFNAR^-/-^ or **(C, D)** NF-κB^-/-^ (plain lines) mice were immunized twice i.m. with gB/SPA06 or gB/SPA10 in the hind leg 21 days apart. **(A, C)** Mice were bled at days 10, 20, and 35 and serum levels of gB-specific IgG, IgG1 and IgG2c were assessed by ELISA. Data are represented as mean ± SEM gB-specific Ab titers for n=5 mice per group. **(B, D)** Spleens were harvested at day 35, and IL-5 and IFN-γ secretion was quantified in supernatants of splenocytes restimulated or not with gB for 72h. The mean cytokine level by gB-stimulated cells after subtraction of medium condition ± SEM are given for n = 4–5 mice per group. One out of two independent experiment is shown. ^‡^ *p* < 0.05 WT gB/SPA06 vs KO gB/SPA06; ^#^ *p* < 0.05 WT gB/SPA10 vs KO gB/SPA10; *p < 0.05.

Type I IFN responses have been linked to the development of adaptive immune responses in numerous studies: they enhance humoral B cell responses, induce T_H_1-type isotype switch (43, 44) and promote the development of T_FH_ cells (45, 46). We thus investigated whether SPA10 exerts its adjuvant effect on B cell responses through type I IFN. WT and IFNAR^-/-^ mice were immunized twice on days 0 and 21 with gB/SPA10 or gB/SPA06. Interestingly, the lack of type I IFN signaling in IFNAR^-/-^ mice did not reduce gB-specific IgG or IgG2c primary nor secondary Ab responses (**Figure 5A**), but increased IgG1 titers. This did not reflect differences in IL-5 secretion by splenocytes, which was similarly reduced following gB/SPA10 or gB/SPA06 (**Figure 5B**). However, IFN-γ was significantly increased in gB/SPA10-immunized IFNAR^-/-^ compared to WT mice (**Figure 5B**).

TLR7 signaling also promotes the expression of pro-inflammatory cytokines through the NF-κB signaling pathway (47), whose contribution to SPA10 adjuvanticity was assessed using NF-κB^-/-^ mice. Ab responses to both SPA06 and SPA10 were strongly dependent on NF-κB activation, as primary and secondary gB-specific IgG, IgG1 and IgG2c titers remained low or undetectable in NF-κB^-/-^ mice (**Figure 5C**). Accordingly, IL-5 and IFN-γ secretion by splenocytes was reduced (**Figure 5D**), indicating that NF-κB activation is required for the adjuvanticity of both SPA06 and SPA10. Thus, SPA06 and SPA10 adjuvanticity does not require type I IFN but is highly dependent on NF-κB activation.

## Discussion

Here, we show that cationic liposomes enhance antibody responses through GC B and T_FH_ cell expansion, and that the incorporation of a TLR7/8 ligand markedly prolongs these responses. Whereas the kinetics of GC B and T cell responses induced by SPA06 appeared similar to those induced by Alum, (35), the oil-in-water emulsions MF59-like Addavax (48) or GLA-SE (38) as reported in other studies, SPA10 induced remarkably long-lasting T_FH_ and GC B cell responses. This phenomenon is associated to the sustained recruitment of highly activated innate cells (mostly monocytes, neutrophils and Mo-DCs) at the site of immunization and dLNs. We ascribe the unique duration of T_FH_/GC responses to SPA10 to this prolonged local activation, and more specifically to the retention of TLR7/8 by the cationic lipids. That a prolonged DC uptake, activation and recruitment to dLNs is associated with sustained CD4^+^ T and GC B cell responses has been already described after immunization with the cationic liposome adjuvant CAF01 (38, 49).

CAF01 was shown to form a long-lasting depot at the site of immunization, in which the antigen is retained and slowly picked-up by incoming antigen-presenting cells (50). Although recognized long ago as a key factor of adjuvanticity, whether this “depot effect” is required for adjuvanticity remains controversial, notably regarding Alum (51). Here we report that while liposomes do not alter the kinetics of the local transient inflammation elicited with antigens alone, the incorporation of a TLR7/8 agonist in the formulation markedly prolonged T and B cell responses. The sustained delivery of both the immunomodulator and the antigen to the dLNs appears as critical for optimal adjuvanticity. This is supported by recent studies showing an enhanced adjuvanticity of 3M-052 when incorporated in liposomes or oil-in-water emulsions (10, 27, 30) or even adsorbed to Alum (32, 33), whereas free TLR7/8 ligand (R848) showed no or moderate adjuvanticity (16–19, 21). This effect was not restricted to the 3M-052 ligand as the adsorption of another TLR7/8 agonist compound to alum (28) potentiates immune responses to glycoconjugate (52) or pertussis vaccines (53). In contrast, Wilkinson *et al.* recently reported that the lipid conjugation of the TLR7 agonist resiquimod to CAF01 does not potentiate immune responses, despite a depot at the site of immunization (54). However, CAF01 already induces strong T_H_1 responses (55) and contains the potent TDB immunomodulator which promotes APC activation through the C-type lectin receptor Mincle (56). This therefore suggests a lack of synergy between C-type lectin- and TLR7-activated pathways, the strong potency of CAF01 masking the effects of resiquimod.

Unexpectedly, despite extended GC B cell responses, the magnitude of SPA10-induced gB-specific IgG antibodies did not differ compared to SPA06, except for a strong shift from IgG1 to IgG2a. This may reflect dose effects or the intrinsic adjuvanticity of cationic lipids, possibly triggering already maximal B cell responses. That a booster dose of gB/SPA10 on day 21 resulted only in a modest increase of antibody responses (**Figures 5A, C**) is likely explained by the ongoing GC reaction. Experiments including analyses at later time-points and dose-response experiments would be required to address these two findings, which were unexpected and thus not included in our ethical clearance application/authorization.

SPA10 upregulated TLR7-signaling related genes, resulting in the induction of type I IFN response. Although we did not assess intracellular trafficking of the liposomes, assuming that cationic liposomes would enter cells by absorptive endocytosis (57), these results indirectly confirm that the liposomes enter the cells and that 3M-052 activates the TLR7 receptor located within the endosome. Despite the strong induction of type I IFN-α responses by SPA10, the abrogation of IFN-α signaling pathway in IFNAR^-/-^ mice did not affect antibody responses to gB, anti-gB IgG1 being even increased compared to WT mice. These contrast with reports showing that anti-IFN-α treatment before OVA/R848 immunization inhibited IgG2a secretion by 80–90% and prevented IgE inhibition (14). Anti-TNF-α or anti-IL-12 lead to similar results, indicating redundant mechanisms, most likely NF-κB-dependent, as contributing to the IgG2a switch (14). In this study, adjuvanticity was triggered only by TLR7/8 signaling, whereas in our setting, adjuvanticity is mediated not only by TLR7/8 signaling but also by the liposomes. Indeed, cationic liposomes have immunostimmulatory properties on DCs as DOTAP and DOEPC liposomes were reported to upregulate expression of the co-stimulation markers CD80 and CD86 (58, 59) and DOTAP liposomes were shown to induce secretion of IL-12 and reactive oxygen species (59, 60). Interestingly, DOTAP- and DOEPC-based cationic liposomes were shown to induce the expression of a secreted alkaline phosphatase reporter gene under the control of a NF-κB promoter, downstream of TLR7 or TLR9 signaling and in a dose-dependent manner (61, 62), suggesting that these types of cationic liposomes have the intrinsic capacity of activating TLR7 or TLR9. In accordance with these observations, we show here that SPA06 upregulates expression of CD80 and CD86 by DCs, but also monocytes and Mo-DCs, and induces expression of TLR7 and type I IFN signaling-related genes, although at low levels. Whether cationic liposomes, and more specifically EDOPC-based liposomes as used here, might exert adjuvanticity *via* other TLRs activation remains to be determined. That the expression of CD80 and CD86 was further enhanced by triggering TLR7/8 strongly suggests synergy between TLR7/8 ligand and cationic liposomes. Therefore, it may be that the true effect of TLR7/8 ligand on adjuvanticity of SPA10 is through enhancement of the physical retention of the formulation and sustained activation of APCs rather than through induction of type I IFN-dependent responses. Nevertheless, we cannot exclude that the strong IFN-γ secretion observed in IFNAR^-/-^ mice upon gB/SPA10 immunization might compensate for the lack of IFN-α-associated responses. Although such a negative regulation of IFN-γ by type I IFN has been observed during viral infection in an IFNAR- and STAT1-dependent manner (63), this has never been described in the context of TLR7/8-adjuvanted vaccines.

These promising results add to those of others suggesting that TLR7/8 ligands may exert potent adjuvanticity on both B cells and T cells – when incorporated in formulations resulting into their sustained released into dLNs. Nevertheless, caution should be exerted when translating TLR agonist-induced immune profiles from small animals to humans given strong differences of TLR expression between doses and species (64). In humans, TLR7 expression is mostly restricted to B cells and pDCs (65), while it is present on myeloid cells in mice (66). TLR8 is expressed by myeloid DC, monocyte-derived DCs, monocytes and neutrophils in humans, while it has long been considered as non-functional in mice (8). Studying mice is nevertheless useful to distinguish the effect of adding TLR7/8 agonists to formulations already exerting adjuvant effects – as shown here. It is encouraging that studies in NHPs, which respond to TLR7/8 ligation more similarly to humans than mice, showed that TLR ligands adsorption to alum enhanced humoral responses to HIV-env (67, 68).

Altogether, our data strengthen the attractiveness of cationic liposome-based approaches capable of retaining both antigen and immunomodulators at the site of injection and of eliciting a sustained influx of APCs at the site of injection. It shows that incorporating TLR7/8 ligands may elicit uniquely long-lasting germinal center and follicular T helper cell responses through the prolonged recruitment of antigen-loaded activated APCs toward the dLNs – even when type I IFN responses are impaired. This opens new promising perspectives in the development of vaccines that might be efficient even in immunocompromised patients or at the extremes of age.

## Data Availability Statement

The raw data supporting the conclusions of this article will be made available by the authors, without undue reservation.

## Ethics Statement

The animal study was reviewed and approved by Geneva Veterinary Office.

## Author Contributions

FA, EB, BM-G, P-HL, C-AS designed the study. FA, EB, and BM-G performed the experiments. FA, EB, BM-G, P-HL, and C-AS analyzed and/or interpreted the results. FA and C-AS wrote the manuscript. All authors contributed to the article and approved the submitted version.

## Funding

This study was supported by funding provided by Sanofi Pasteur and research grants of the Center for Vaccinology and Neonatal Immunology.

## Conflict of Interest

The authors declare that this study received funding from Sanofi Pasteur. The funder had the following involvement with the study: SPA06 and SPA10 were developed by Sanofi Pasteur. 3M-052 is proprietary of 3M Pharmaceuticals.

## Abbreviations

Ab, antibody; Ag, antigen; DC, dendritic cell; dLN, draining lymph node; DOEPC, 1,2-dioleoyl-*sn*-glycero-3-ethylphosphocholine; gB, glycoprotein B; GC, germinal center; Mo-DC, monocyte-derived dendritic cell; PMN, polymorphonuclear cells; T_FH_, T follicular helper cells; TLR, Toll-like receptor.
